# The Control of Cancer: Two Books for Non-Medical Readers

**Published:** 1916-07-08

**Authors:** 


### THE CONTROL OF CANCER.

### Two Books for Non-Medical Readers.

Here in Britain practically all medical research is devoted to the pressing therapeutic problems, many of them novel to a large extent, which are presented by the sick and wounded from the armies serving under our Flag. Almost the only country not involved in the great war where scientific research is pursued on a large scale is the United States; and it is interesting to note that public attention is being strongly directed there upon the question of cancer and its control. Apart altogether from the highly technical reports of research workers and laboratories, there is in the United States a marked tendency to the presentation of facts, figures, and deductions from both in such a form that they can be comprehended by any reasonably intelligent member of the general public. As illustrations of this tendency, two bulky volumes have lately reached us from across the Atlantic, though neither appears to be quite new.* One of them is by a surgeon and researcher, the other by a statistician and actuary. Each is a monument of industry, and though there are points upon which they differ, they both bear witness to the focussing of public attention in the United States upon the magnitude of the cancer problem. Furthermore, two Associations have come into existence there which attest the same interest on the part of the public and the medical profession: these are the American Society for the Control of Cancer, and the American Association for Cancer Research.

Three-quarters of Mr. Hoffman's book consists of statistics, gathered from all parts of the globe, upon cancer mortality and other kindred topics. These statistics, compiled by a professional actuary, must prove of the greatest value as a source of reference to cancer workers; but they are scarcely, in themselves, likely to interest the lay reader. Prefixed to them, however, is a carefully constructed argument, based chiefly upon statistical premisses, upon various aspects of the cancer problem. The chief conclusions for which Mr. Hoffman wishes to obtain acceptance is that the admitted great apparent increase in cancer mortality among civilised communities during the last generation or two is, partly at least, a real increase, and cannot be explained away as being wholly apparent. This analysis, written by a layman, is packed full of solid reasoning and valuable information; it is distinctly an acquisition to the literature of cancer.

Of more general interest, however, is Dr. Bainbridge's categorical discussion of every side of the cancer problem. It is written for general readers as well as for the medical profession, and only certain sections on surgical technique as applied to cancer are likely to be skipped as too indigestible for the public's powers of absorption. It may be noted that this author is not in agreement with Mr. Hoffman as to the alleged increase in cancer, but he does not discuss the statistical data with quite the same authority or quite so much in detail as does that author. Incidentally Dr. Bainbridge has little that is good to say about death registration and certification as practised in America, and regards the British figures as much the most reliable of any that can be obtained. Mr. Hoffman seems to pay too little heed to fallacies arising from incorrect registration of the cause of death in cancer cases dictated directly or indirectly by the relatives of the deceased; while Dr. Bainbridge fails to take into account the advances of surgery in recent years, whereby a large number of cancer cases happily never figure in the mortality tables at all.

Dr. Bainbridge is at his best in dealing with cancer research. His readers will soon discover that Dr. E. F. Bashford, of London, looms larger, perhaps, on his horizon than any other worker in this field at the present time. The account of Bashford's successful stand years ago, singlehanded almost, against the German school and its parasitic theory of cancer, and against the International Association for Cancer Research, founded in Germany, emphasises the value of Bashford's adherence to his principles and the independence of his mind and vision at a time when it would have been much easier to swim with the stream. Even more interesting are the sections wherein Dr. Bainbridge describes the tests which have been applied in various scientific institutions to empirical "cancer cures" of many kinds. His *exposé* should go far to convince lay readers that the medical profession is not, as some people believe, hide-bound in slavish adherence to doctrines officially pronounced orthodox; but is willing, nay anxious, to have full and fair tests of any method which promises the very remotest prospect of benefit to those suffering from malignant disease. His case is put with a logical precision and a clarity of expression than which nothing could be more admirably adapted for the end in view, the instruction of the intelligent public.

There are one or two small points which should be rectified in any future edition. Mr. W. E. Miles, of the Cancer Hospital, London, has been confused with Mr. A. Miles, of the Royal Infirmary, Edinburgh. The statement that in 1911 a scare of cancer propagation by eating fish bade fair to paralyse the fish industry of Great Britain is at the very least an exaggeration: we have no recollection of even an incident of the sort, much less of a general scare. Our biggest bone of contention should perhaps be picked rather with the publishers than with Dr. Bainbridge, who may be quite innocent in the matter. This is the inclusion at the end of the volume of an advertisement of a book by a man whose reputation amongst his colleagues is distinctly bad. Apart from this, we have little but praise for a contribution to the literature of the cancer problem which we hope will achieve a wide circulation.

* *The Cancer Problem.* By W. Seaman Bainbridge, M.D. Macmillan. 1915. Pp. 534. 17s. net.
*The Mortality from Cancer throughout the World.* By F. L. Hoffman. 1915. Pp. 826. Prudential Press, New Jersey.

